# Stromal upregulation of lateral epithelial adhesions: Gene expression analysis of signalling pathways in prostate epithelium

**DOI:** 10.1186/1423-0127-18-45

**Published:** 2011-06-22

**Authors:** Karen F Chambers, Joanna F Pearson, Davide Pellacani, Naveed Aziz, Miodrag Gužvić, Christoph A Klein, Shona H Lang

**Affiliations:** 1 Yorkshire Cancer Research Unit, Dept. Biology, University of York, Heslington, York, (YO10 5YW), UK; 2 Genomics Laboratory, Technology Facility, Dept. Biology, University of York, Heslington, York, (YO10 5YW), UK; 3 Division of Oncogenomics, Department of Pathology, University of Regensburg, Regensburg, Germany

## Abstract

**Background:**

Stromal signalling increases the lateral cell adhesions of prostate epithelial cells grown in 3D culture. The aim of this study was to use microarray analysis to identify significant epithelial signalling pathways and genes in this process.

**Methods:**

Microarray analysis was used to identify genes that were differentially expressed when epithelial cells were grown in 3D Matrigel culture with stromal co-culture compared to without stroma. Two culture models were employed: primary epithelial cells (ten samples) and an epithelial cell line (three experiments). A separate microarray analysis was performed on each model system and then compared to identify tissue-relevant genes in a cell line model.

**Results:**

TGF beta signalling was significantly ranked for both model systems and in both models the TGF beta signalling gene SOX4 was significantly down regulated. Analysis of all differentially expressed genes to identify genes that were common to both models found several morphology related gene clusters; actin binding (DIAPH2, FHOD3, ABLIM1, TMOD4, MYH10), GTPase activator activity (BCR, MYH10), cytoskeleton (MAP2, MYH10, TMOD4, FHOD3), protein binding (ITGA6, CD44), proteinaceous extracellular matrix (NID2, CILP2), ion channel/ ion transporter activity (CACNA1C, CACNB2, KCNH2, SLC8A1, SLC39A9) and genes associated with developmental pathways (POFUT1, FZD2, HOXA5, IRX2, FGF11, SOX4, SMARCC1).

**Conclusions:**

In 3D prostate cultures, stromal cells increase lateral epithelial cell adhesions. We show that this morphological effect is associated with gene expression changes to TGF beta signalling, cytoskeleton and anion activity.

## Background

Tissue morphogenesis is controlled by a variety of factors including local growth factors, extracellular matrix, cell adhesion molecules and the cytoskeleton. Cadherins and tight junctions have a major role in establishing and maintaining intercellular adhesion [1,2]. E-cadherin initiates intercellular contacts, forms homophilic adhesions and links to the actin cytoskeleton through β-catenin. The spatial control of cadherin clusters by the actin cytoskeleton is important for stable adhesions [3,4]. In adult polarised epithelial tissues adherens junctions are further associated with tight junctions leading to the formation of the apical junctional complex. Tight junctions provide epithelial cells with a paracellular diffusion barrier that is critical for normal tissue function and maintenance of polarity [5,6]. The shape of an epithelial cell is related to its function, to adhesion molecules and to their interaction with an organised actin cytoskeleton. The mechanisms controlling lateral cell adhesions in an adult tissue are not fully understood. An understanding of the molecular pathways which govern junctional proteins and actin cytoskeleton organization are required to further our understanding of normal tissue and the development of diseases.

We have previously modelled prostate epithelial morphogenesis using 3D Matrigel culture [7]. Primary epithelial cells, grown in 3D Matrigel, form hollow acinus-like gland structures and co-culture of these structures with stromal cells leads to increased polarisation and increased lateral cell adhesions between the epithelial cells. Significantly, this result contradicts the role of stroma in epithelial mesenchymal transition [8] and suggests that the role of stroma in 3D culture supports a role for stroma in the maintenance of tissue integrity. In support of this, mouse modelling of the prostate also demonstrated the requirement for stroma to induce architectural organisation [9]. Our recent work has demonstrated that stromal derived TGFβ2 can increase the co-localisation of E-cadherin with the actin cytoskeleton and decrease paracellular permeability (paper in submission). The control of any biological process is highly complex, involving many signalling pathways. To identify epithelial genes and signalling pathways that are controlled by stromal cells in 3D culture, we employed microarray analysis and bioinformatics. Micorarray information derived from limited numbers of cell lines does not always represent information derived from tissue. However cell lines provide useful reproducible model systems in the laboratory with which to understand complex biological processes. Therefore we aimed to combine microarray information derived from both primary and cell line cultures, to identify genes that are relevant to tissues, but could be further investigated in cell line model systems. Key pathways and gene clusters were identified that were associated with TGF beta signalling, cytoskeleton, ion channel/ion transporter activity and developmental pathways.

## Methods

### Primary culture

The use of human prostate tissue to grow primary cultures and patient consent procedures were approved by York Research Ethics Committee, (YREC Reference 91/7/6) and Hull and East Riding Local Research Ethics Committee (REC Reference Number 07/H1304/121). Tissue was obtained from York District Hospital, York and Castle Hill Hospital, Hull, UK. All patients who provided tissue gave their written consent. Tissues were given a unique identification number which was stored with the consent forms at participating hospitals, whilst documentation of tissue processing, experimentation and storage occurred at the YCR Cancer Research Laboratory.

Primary cultures were prepared as described before [7]. Briefly, prostatic tissue was digested with collagenase and trypsin, and differential centrifugation was used to enrich for epithelial and stromal fractions. The enriched stromal fraction was resuspended in stromal cell growth medium (RPMI1640 supplemented with10% FCS and 1% antibiotic/antimycotic solution) and cultured routinely in 75-ml tissue culture flasks. The epithelial fraction was resuspended in keratinocyte serum-free medium (KSFM) supplemented with 5 ng/ml epidermal growth factor and 1% antibiotic/antimycotic solution (medium subsequently referred to as KSFM.

### 3D Matrigel culture

BPH-1 cells (benign prostate cell line), primary human benign prostate epithelial cultures and primary human benign prostate stromal cultures were cultured in 3D as described previously [7,10].

Briefly, Primary stromal cultures (passage 1 to 3) were seeded prior to co-culture in 0.4 μm Millicell-PCF inserts (Millipore, Livingston, UK), 2 × 10^4 ^cells/insert in RPMI supplemented with 10% FCS, until confluent. Epithelial cells were seeded at 5 000 cells/ml in KE2 (KSFM, 2% FCS, 2 mM L-glutamine, 10 nM dihydrotestosterone and 10 nM β-estradiol) and 4% (v/v) Matrigel. Inserts were then washed with PBS and added to epithelia plus Matrigel or blank wells, with KE2. The inserts were replaced 4, 8 and 12 days after cell seeding with fresh inserts of pre-seeded stroma. Medium was replenished at the same time through the removal of 0.5 ml spent media and the addition of 0.5 ml fresh KE2 supplemented with 4% Matrigel. Spheroids for RT-PCR were isolated from the Matrigel using BD Cell recovery solution (Becton Dickinson, Plymouth, UK).

### mRNA isolation, cDNA synthesis and global amplification from a single spheroid for Operon array

Ten primary epithelial cultures (passage 1) were grown in Matrigel, with or without primary prostate stroma for 14 days, the optimum time of primary spheroid formation [7]. Single acini were isolated by "picking" spheroids with a pipette from a PBS/Matrgel suspension on a blocked dish (0.3% BSA). RNA was prepared from the single spheroid amplification step according to a previous method [11].

### Operon array analysis

Array pre-processing and significance analysis was performed using GeneSpring GX 10 software (Agilent Technologies, Inc). Arrays were filtered on expression between the 20th and 100th percentile of the raw data. Normalization was performed by scaling and baseline transformation to the median of all samples. The experiment was analysed as a reference design. Differentially expressed genes were identified by using a paired t-test with asymptotic p-value computation and no multiple testing correction where significance level was set at p > 0.05. Genes that were > 1.1 fold up- or down-regulated between groups were selected, this was then referred to as the 'primary 1.1 fold gene list. [Accession number; E-MEXP-2994].

### Affymetrix microarray and analysis

Three replicate cultures of BPH-1 (passage 47) were grown in 24 well plates with or without stroma (a mix of 3 different stromal cultures at passage 2) for 7 days in KE2 media. A time point of 7 days was chosen, since BPH-1 cells grow faster than primary cultures. On average, 7 day old BPH-1 acini are the same size as 14 day old primary acini. 3D acini produced by BPH-1 cells are predominantly homogeneous, therefore individual acini were not isolated, RNA was prepared from whole cultures and an Affymetrix array was performed. RNA was prepared using Illustra RNA Spin mini kit (GE Healthcare, Little Chalfont, UK). RNA samples were analysed using Affymetrix Human Genome U133 Plus 2.0 chips (Affymetrix Inc., Santa Clara, CA). Each array contains more than 54,676 probe sets that represent more than 47,000 transcripts. The RNA hybridisation of all Affymetrix U133 Plus 2.0 arrays was performed at TF facility (University of York). The cRNA synthesis of the samples was carried out according to the manufacturer's protocol. The fluorescence intensity for each chip was captured with an (Affymetrix GeneChip Scanner 3000). Affymetrix Microarray Suite version 5.0 (MAS 5.0, Affymetrix) was used to quantitate each chip. The raw data (CEL) files, were loaded into the DNA-chip analyser software (dChip) version Feb 2009 [12]. Normalisation was carried out using Invariant Set Normalisation method and probe expression values were then calculated using the perfect match (PM)-only model according to Chambers et al., 2009 [13]. Unsupervised hierarchical sample clustering was performed on a list of probes derived by filtering probes using the criteria of standard deviation divided by the mean between 0 and 1000 across the samples and the samples clustered into two separate groups, indicating reproducibility of the data. Three comparison criteria were applied to the data to detect differentially expressed genes by model based expression: 1) the fold change between the group means was chosen to exceed 1.5 fold 2) absolute difference between the two groups means > 50 to eliminate the very low expressing genes that have intensity close to background levels and 3) a p-value of 0.05 for Welch's modified 2-sample paired t-test, adjusted to compensate for multiple testing using False discovery rate (FDR). In dChip, the FDR was estimated by a 1000 permutations.

Raw data was processed using the Affymetrix GCOS 1.2 software. After hybridization and scanning, probe cell intensities were calculated and summarized for the respective probe sets by means of the MAS5 algorithm. To compare the expression values of the genes from chip to chip, global scaling was performed, which resulted in the normalization of the trimmed mean of each chip to target intensity (TGT value) of 500 as detailed in the statistical algorithms description document of Affymetrix (2002; http://www.affymetrix.com). Each sample and hybridization underwent a quality control evaluation mainly checking for adequate scaling factors (1-3 for all samples), percentage of probe sets reliably detected (between 40-60% present call), and optimal 3'/5' hybridization ratios (~1) for the housekeeping genes (e.g., GAPDH), poly(A) spike-in controls, and the prokaryotic controls (bioB, bioC, bioD and cre). MAS5 normalised data were collected and analyzed using the GeneSpring GX10 Expression software (Agilent Technologies, USA). Differentially expressed genes were identified by using a two-class t test where significance level was set at p > 0.05. Genes that were > 1.1 fold up- or down-regulated between groups were selected. [Accession number; E-MEXP-2990].

### Pathway Express

Functional analysis was performed on the 1.1, p < 0.05 probe lists using Pathway-Express http://vortex.cs.wayne.edu[14]. Pathway Express ranks pathways using classical impact factors but deepens the statistics by adding into the analysis, the magnitude of gene expression change and the position and interaction within the pathway. The gamma p-value is provided by the impact analysis.

### Analysis of common genes from the primary culture and cell line arrays

Two separate fold change lists were generated using Genesping. Both lists were generated using the same fold change of 1.1 fold and a p < 0.05. The first list describes all the probes changing in BPH-1 acini cultured with stroma compared to control BPH-1 spheroids cultured without stroma. The second list describes the probes changing from single primary prostate acini cultured with and without stroma. To compare the genes that were the common between both lists and thus compare the BPH-1 cell line acini to primary acini the Entrez gene IDs were used. The probe IDs could not be used as Operon arrays were used for the single primary acini culture and Affymetrix arrays were used for the whole population BPH-1 cell culture. The up-regulated and down-regulated Entrez IDs between the two lists were compared using the following function in excel VLOOKUP(B3,A:A,1,FALSE). The Entrez IDs that matched were copied into a txt. file and the gene names found using http://david.abcc.ncifcrf.gov/conversion.jsp.

### RT2 Profiler PCR Array

RNA was prepared from BPH-1 spheroids grown in 24 well plates using Illustra RNA Spin mini kit (GE Healthcare, Little Chalfont, UK) and grown with and without stroma (a mix of 3 different stromal cultures at passage 2, different cultures to the array). Reverse transcription was performed with RT2 PCR array First Strand Kit (SABiosciences). RT2 profiler PCR array for the human TGFB BMP signaling pathway were prepared as per manufacturers protocol. Target cDNA levels were detected using the ABI prism 7300 sequence detection system (Applied Biosciences) and normalised to HPRT, B2M, RPL13A and ACTB using the DDCt Data analysis method. The real time PCR conditions were as follows: 1 cycle at 95°C for 10 min, 40 cycles at 95°C for 15 s, and 60°C for 1 min. 49 genes appearing on the TGF beta PCR array were not differentially expressed according to the microarray data.

### Real-time quantitative PCR (QRT-PCR)

RNA was prepared from spheroids using Illustra RNA Spin mini kit (GE Healthcare, Little Chalfont, UK). Reverse transcription was performed with random hexamers (SuperScript™ II Reverse Transcriptase kit, Invitrogen). Quantitative real time PCR oligonucleotide primers (Additional file 1, Table S1) and fluorigenic Taqman probes were designed using Primer Express 3.0 software (ABI Prism; Applied Biosystems). Reactions used Taqman one-step mastermix kit (Applied biosystems), 400 nM of each gene specific primer, 100 nM each probe and 100 ng total cDNA (25 μl volume). Target mRNA levels were detected using the ABI prism 7700 sequence detection system (Applied Biosciences) and normalised to HPRT using the relative quantification method. The real time PCR conditions were as follows: 1 cycle at 50°C for 2 min, 1 cycle at 95°C for 10 min, 40 cycles at 95°C for 15 s, and 60°C for 1 min. Assays consisted of three technical replicates.

## Results

### Primary epithelial gene expression changes and pathways induced by stromal secreted factors in 3D culture

**Figure 1:**
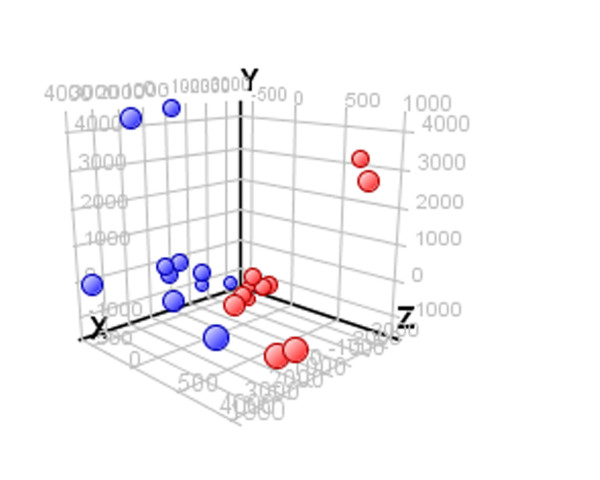
**Primary component analysis to show clustering of genes from acini cultured with stroma (blue) compared to acini cultured without (red)**.

To identify the pathways and genes that control lateral epithelial adhesions in prostate cells we compared the RNA expression patterns between 3D acini grown with and without stromal co-culture in 3D. To identify tissue relevant genes and not just gene changes associated with a single cell line we chose to perform our experiments on primary epithelial and primary stromal cell cultures. Most primary epithelial cells grown in 3D gels develop into clusters of spherical-acinus-like structures however some cells undergo limited divisions and others do not divide at all. To avoid immature acini or single cells contaminating our results we isolated individual acini (ten primary epithelial cell cultures) and hybridized their transcriptomes on an Operon microarray, which is known to be robust for low cell numbers [11]. Comparison of RNA expression patterns from 3D acini cultured with and without stroma identified 1574 probe sets with significant differential expression in between the two groups (paired t test, p < 0.05). We used principal component analysis to demonstrate that the genes identified could be used to distinguish between stromal co-culture or not within our data set (Figure 1). The ten most upregulated and down regulated genes are listed in Table 1. To identify key functional categories within the differentially expressed genes we performed Pathway Express analysis (Table 2). Thirteen pathways were significantly ranked. The most highly ranked pathway was cell adhesion molecules, which predominantly indicated the upregulation and down regulation of claudin (CLDN) and integrin (ITG) isoforms.

**Table 1:** The ten most upregulated and down regulated primary epithelial genes in response to stroma, in 3D culture

Gene Symbol	Gene Title	Fold change	Probe ID
FGFBP1	fibroblast growth factor binding protein 1	3.078	H300006842
OR4N5	olfactory receptor, family 4, subfamily N, member 5	2.993	opHsV0400002653
KLRC4	killer cell lectin-like receptor subfamily C, member 4	2.735	H300021805
TESSP1	testis serine protease 1	2.656	opHsV0400002778
KT3K	Ketosamine-3-kinase	2.528	H200004088
KLRC1	killer cell lectin-like receptor subfamily C, member 1	2.420	H200005914
SNX12	sorting nexin 12	2.330	H200009199
PABPC1	poly(A) binding protein, cytoplasmic 1	2.323	H200014179
DNMBP	Dynamin binding protein	2.282	opHsV0400003719
MSH4	mutS homolog 4	2.265	H200011946
IL23R	interleukin 23 receptor	-2.489	H300020818
DYNC2LI1	dynein 2 light intermediate chain isoform 1	-2.553	opHsV0400000277
KRT6B	keratin 6B	-2.560	H300014335
SAA1	serum amyloid A1	-2.600	opHsV0400005893
TOP1	topoisomerase (DNA) I	-2.844	opHsV0400006965
PTPLAD1	protein tyrosine phosphatase domain containing 1	-2.859	H300003447
SEMA3C	sema domain, secreted 3C	-3.412	H200008006
KRT23	keratin 23	-3.552	H300014341
C3	complement component 3	-3.553	H20000880
MAGI1	membrane associated guanylate kinase, WWW and PDZ domain containing 1	-4.678	H200002533

Values are the mean fold expression changes of ten primary epithelial cell cultures, abstracted from Operon datasets. All genes showed significant differential expression (p < 0.05). Positive values indicate up regulation, negative values indicate down regulation of epithelial genes cultured with stroma compared to without stroma. Hypothetical or unassigned genes were removed from the list.

**Table 2:** Significantly induced pathways associated with the response of primary epithelia to stroma co-culture in 3D

Rank	Pathway	Impact Factor	no. genes/ genes in pathway	Upregulated genes	Downregulated genes
1	Cell adhesion molecules	246.278	9/133	CLDN22, CLDN6, HLA-DPB1, ITGB2, NRXN3, PTPRC	CD22, CLDN7, ITGA6

2	Phosphatidyl inositol signalling	27.591	4/77	PRKCB1	DGKG, INPP4B, PLCD3

3	Antigen processing and presentation	20.271	6/88	HLADPB1, KLRC2, KLRC4, IFNA21, KIR3DL2	NFYB

4	Adherens junction	10.67	2/75	ACVR1B	PTPRB

5	Type II diabetes mellitus	7.677	2/44	CACNA1C, CACNA1E	

6	Long term depression	5.7	6/76	GRM1, NPR2, PRKCB1	CRHR1, HRAS, PLA2G6

7	Gap junction	5.297	8/96	GRM1, HTR2, NPR2, PRKCB1, TUBB6	HRAS,PRKACB

8	Tight junction	5.253	10/135	CLDN22, CLDN6, MYH10, MYH6, PRKCB1	CLDN7, HRAS, MYH14, PP2R4, PRKCQ

9	Melanoma	5.206	4/71	FGF11, FGF2, FGF18	HRAS

10	Basal cell carcinoma	5.174	1/55	FZD2	

11	TGF beta signalling	4.763	3/89	ACVR1B, DCN	ZFYVE9

12	Glioma	4.756	4/64	PRKCB1	CAMK2D, HRAS, SHC2
13	MAPK signalling pathway	4.747	19/265	CACNA1C, CACNA1E, CACNA1S, CACNB2, ACVR1B, FGF11, FGF2, FGF18, FOS, JUN, PAK1, PRKCB1	CRKL, HRAS, MAP3K7IP2, PLA2G6, PRKACB, PRKX

The 'primary 1.1 fold gene list' was loaded into Pathway Express to determine the significant epithelial pathways associated with co-culture with stroma in 3D. The 1.1 fold gene list was derived from all ten primary epithelial cell cultures. All significantly (p < 0.05) ranked pathways are shown (91 ranked in total).

### Heterogeneity in gene expression between primary epithelial cell samples

**Figure 2:**
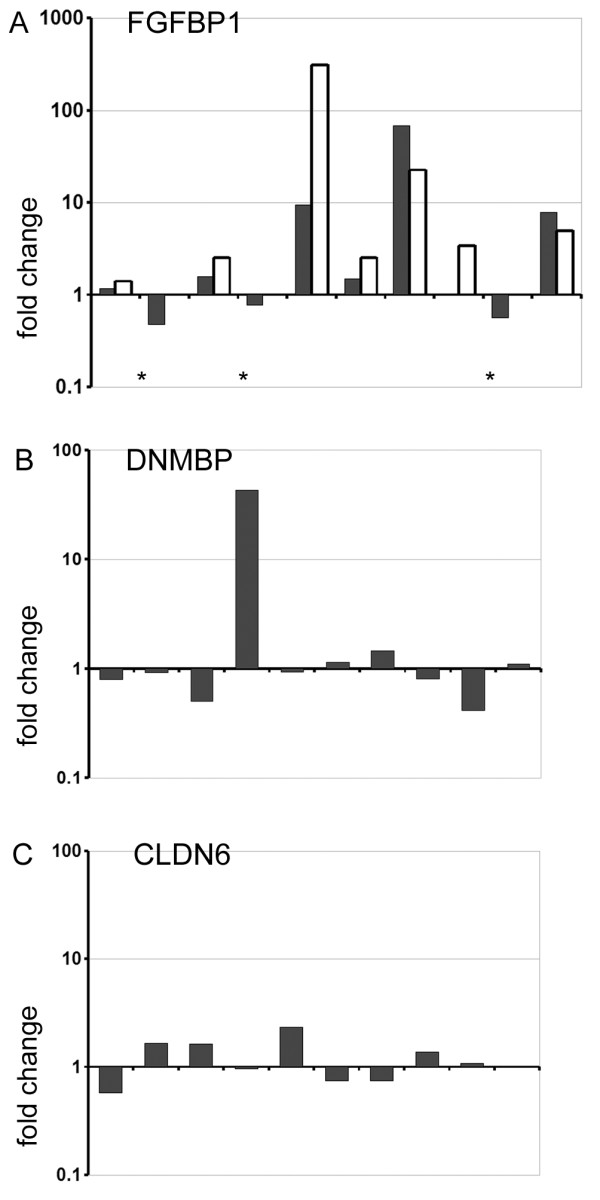
**Evidence of heterogeneity in gene expression between different primary culture samples**. Primary epithelial cells were grown for 14 days in Matrigel with stromal co-cultures and compared to cultures grown without stroma. RNA was prepared from single acini. Data is shown for all ten matched primary epithelial cell cultures. A) the mean fold change in gene expression levels for FGFBP1 measured by Operon microarray (black bars) or QRT-PCR (open bars). B) the mean fold change in gene expression levels for DNMBP measured by Operon microarray. C) the mean fold change in gene expression levels for CLDN6 measured by Operon microarray. Changes in gene expression detected by Operon were from the 1.1 fold gene list (p < 0.05).* insufficient sample available for QRT-PCR

To verify the Operon microarray data we selected FGFBP1 (fibroblast growth factor binding protein 1), since the average expression of this genes was highly upregulated in the presence of stroma. Using matched patients samples to the microarray, we performed quantitative RT-PCR (QRT-PCR). QRT-PCR confirmed the upregulation of FGFBP1 in six primary epithelial samples in response to stromal co-culture (Figure 2A). One epithelial sample showed no change in gene expression by array data but upregulation by QRT-PCR. Three samples showed down-regulation from the array data, but insufficient material prevented QRT-PCR analysis. Therefore, we observed good confirmation of the microarray analysis by QRT-PCR, but analysis of individual patient data sets indicated that different epithelial cultures had very variable expression of FGFBP1. Further verification of DNMBP expression (Figure 2B) and CLDN6 expression (Figure 2C) indicated that the culture/patient heterogeneity was not limited to FGFBP1. Although average gene expression of DNMBP and CLDN6 was upregulated (Tables 1 and 2), analysis of individual cultures/patient samples indicated that DNMBP was upregulated in only 4/10 samples and CLDN6 in 5/10 samples. It was evident that the mean fold change in expression was dependent predominantly on a low number of high differential expressors and was not typical of the whole population of epithelial samples.

### BPH-1 cell line gene expression changes and pathways induced by stromal secreted factors in 3D culture

To overcome the problems of heterogeneity we decided to analyse a prostate epithelial cell line, BPH-1, which can also grow into acinus-like spheroids in 3D culture and demonstrates increased lateral adhesions, in response to stroma [10]. We performed a second microarray experiment to compare the RNA expression patterns between 3D BPH-1 acini grown with and without stromal co-culture. The cell line model array would then inform the primary culture model, allowing us to identify shared differentially expressed genes and pathways. This would provide a dataset that was relevant to human adult tissues, but within a reproducible cell line model. Common genes may also be more fundamental to adhesion and therefore of greater importance to future functional studies.

Three technical replicates of BPH-1 cells were cultured in 3D with and without primary stroma (an identical mix from three different patients), using identical culture conditions to the primary cell model. 7843 probe sets were differentially expressed between the two experimental groups (p < 0.05). Table 3 lists the most differentially expressed genes and table 4 lists the pathways with an impact factor greater than 4 (62 pathways were ranked in total). The highest ranking pathway was ECM-receptor interactions. Eleven of the ranked pathways were significant and, of these, only TGF beta signalling was listed for both primary cells and cell lines datasets. KRT6B was highly down regulated in both models (table 1 and 3).

**Table 3:** The ten most upregulated and down regulated BPH-1 genes in response to stroma, in 3D culture

Gene Symbol	Gene Title	Fold change	Probe ID
MAP7D2	MAP7 domain containing 2	2.866	228262_at
IGFBP3	insulin-like growth factor binding protein 3	2.613	212143_s_at
IGFBP3	insulin-like growth factor binding protein 3	2.600	210095_s_at
STON1	stonin 1	2.527	213413_at
BEAN	brain expressed, associated with Nedd4	2.446	214068_at
FBXO32	F-box protein 32	2.290	225803_at
GNG7	guanine nucleotide binding protein (G protein), gamma 7	2.277	228831_s_at
NOTCH3	Notch homolog 3 (Drosophila)	2.264	203238_s_at
MB	myoglobin	2.258	204179_at
CLIC3	chloride intracellular channel 3	2.225	219529_at

KRT6A /// KRT6B /// KRT6C	keratin 6A /// keratin 6B /// keratin 6C	-3.099	214580_x_at
NNMT	nicotinamide N-methyltransferase	-3.126	202237_at
KIAA1199	KIAA1199	-3.183	212942_s_at
KRT6A	keratin 6A	-3.215	209125_at
HSD17B2	hydroxysteroid (17-beta) dehydrogenase 2	-3.450	204818_at
KRT14	keratin 14	-3.460	209351_at
SERPINB3 /// SERPINB4	serpin peptidase inhibitor, clade B (ovalbumin), member 3 /// serpin peptidase inhibitor, clade B (ovalbumin), member 4	-8.311	210413_x_at
SERPINB3	serpin peptidase inhibitor, clade B (ovalbumin), member 3	-9.406	209719_x_at
SERPINB3	serpin peptidase inhibitor, clade B (ovalbumin), member 3	-12.240	209720_s_at
SERPINB4	serpin peptidase inhibitor, clade B (ovalbumin), member 4	-17.045	211906_s_at

*Values are mean fold expression changes of three BPH-1 cultures abstracted from Affymetrix datasets. All genes showed significant differential expression (p < 0.05). Positive values indicate upregulation, negative values indicate down regulated genes in the epithelial cells. Hypothetical or unassigned genes were removed from the list.

**Table 4:** Significant pathways associated with the response of BPH-1 cells to stroma in 3D culture

Rank	Pathway Name	Impact Factor	no. genes/ genes in pathway	Upregulated genes	Downregulated genes
1	ECM-receptor interaction	14.5*	10/84	COL6A1, LAMB2	CD44, CD47, FN1, ITGA2, LAMB1, THBS1

2	Adherens junction	11.94	1/78		MET

3	Haematopoietic cell lineage	9.93*	8/87		CD44, CD55. CD59, IL1R2, IL4R, IL7R, ITGA2, ITGA6

4	Small cell lung cancer	7.96*	7/86	LAMB2	FN1, ITGA2, ITGA6, LAMB1, MYC

5	Basal cell carcinoma	7.83	2/55	WNT10A, WNT4	

6	Pathways in cancer	7.27*	13/330	DAPK2, LAMB2, WNT10A, WNT4	ETS1, FN1, ITGA2, ITGA6, LAMB1, MET, MYC, RUNX1

7	Focal adhesion	7.02*	10/203	COL6A1, LAMB2	FLNB, FN1, ITGA2, LAMB1, MET, THBS1

8	TGF-beta signalling pathway	6.61*	5/87	INHBB	FST, MYC, THBS1, ID1

9	Axon guidance	6.48*	7/129	EFNB3, NFATC4, NTN1, SEMA3C	MET, PLXNC1, SRGAP1

10	Jak-STAT signalling pathway	6.32*	7/155		IL13RA2, IL24, IL4R, IL7R, MYC, OSMR, SOCS3

11	Calcium signalling pathway	4.93	2/182		EDNRA, HRH1

12	Bladder cancer	4.74*	3/42	DAPK2	MYC, THBS1

13	B cell receptor signalling pathway	4.62	2/65	BLNK, NFATC4	

14	Complement and coagulation cascades	4.59*	4/69	C3	CD55, CD59, F3

15	Cytokine-cytokine receptor interaction	4.56*	9/263	INHBB	CXCL5, IL1R2, IL24, IL4R, IL7R, MET, OSMR, PPBP

16	Regulation of actin cytoskeleton	4.37	5/217	CYFIP2	FN1, ITGA2, ITGA6, SSH1

17	Regulation of autophagy	4.32	1/35	GABARAPL1	

18	Melanoma	4.25	1/71		MET

19	Wnt signaling pathway	4.2	4/152	NFATC4, WNT10A, WNT4	MYC

20	Tight junction	4.11	2/135	JAM2, TJP3	

The 'BPH1 1.1 fold gene list' was loaded into pathway express. All pathways with an impact factor > 4 are listed. * significant pathways, p < 0.05. The 1.1 fold gene list was derived from all three BPH-1 cultures.

### The TGF beta signalling pathway is significant for primary and BPH-1 arrays

**Figure 3:**
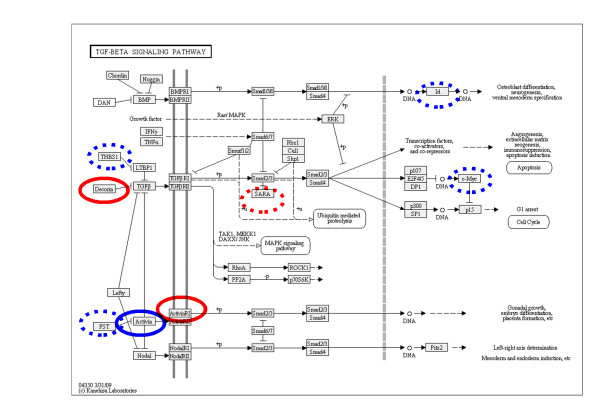
**Genes identified in the TGF beta pathway**. The Kegg pathway for TGF beta signalling illustrating the significant genes identified by pathway express. Genes circled in red indicate genes indicate genes from the primary cell microarray dataset (Operon) and those in blue from the BPH-1 cell microarray dataset (Affymetrix). Continuous lines are upregulated genes whereas broken lines are down-regulated.

Figure 3 shows the Kyoto Encyclopedia of Genes and Genomes (Kegg) pathway for TGF beta signalling [15-17] and illustrates the significant genes found by Pathway Express for both primary and cell line microarray datasets. No gene was expressed by both arrays on the Kegg pathway. The primary cultures showed upregulation of ACVR1B (activin receptor type-1B) and DCN (decorin) and down regulation of SARA (ZFYVE9) in response to stromal co-culture. BPH-1 cells showed upregulation of INHBB (inhibin beta chain) and down regulation of FST (follistatin), MYC, THBS1 (thrombospondin 1) and ID1.

**Figure 4:**
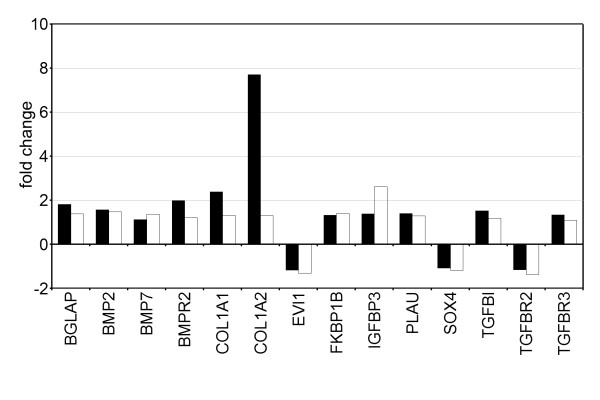
**Validation of human TGF beta/ BMP signalling pathway genes**. Average changes in gene expression between 3D BPH-1 acini cultured with stroma compared to without stroma detected by Affymetrix (white bars) and QRT-PCR techniques (black bars). Changes in gene expression detected by Affymetrix were from the 1.1 fold gene list (p < 0.05).

To verify the BPH-1 microarray data and in particular genes associated with TGF beta signalling pathway, we used a commercial PCR array for the 'human TGF beta/BMP signaling pathway'. The differential expression of fourteen genes was verified (Figure 4); BGLAP (osteocalcin), bone morphogenic proteins and receptors (BMP, BMPR), type 1 collagens (COL1), TGF beta induced (TGFBI) and TGF beta receptors 2 and 3, IGFBP3 (Insulin-like growth factor binding protein 3), PLAU (Urokinase-type plasminogen activator), FKBP1B (peptidyl-prolyl cis-trans isomerase), SOX4 and EVI1 (Ecotropic viral integration site 1). THBSP1, ACVR1B, DCN and ZFYVE9 did not appear on the QRT-PCR. Twenty one differentially expressed genes in the microarray data were not confirmed by QRT-PCR (results not shown). The low correlation between the microarray data and QRT-PCR using the same cell line is likely due to stromal heterogeneity (the experiments were performed with different stromal culture mixes). However, the genes in agreement will represent more robust candidates

### Identification of common genes which are upregulated or down regulated in both primary and BPH-1 microarray datasets

**Figure 5:**
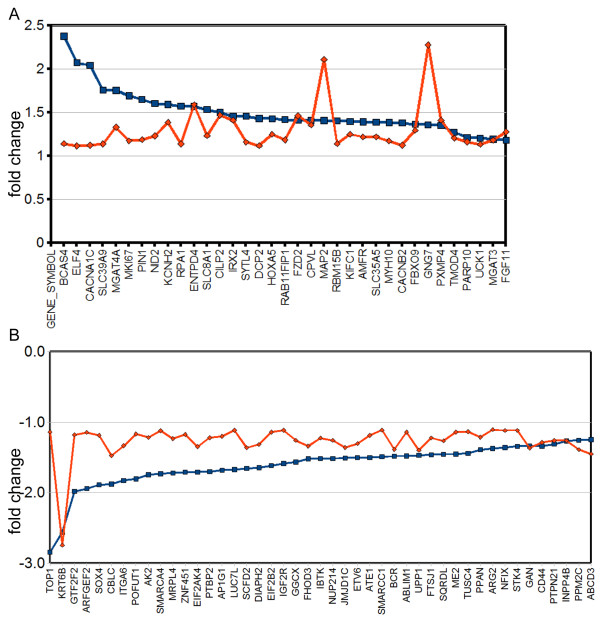
**Identification of common genes that were up regulated or down regulated in both cell line and primary epithelial models in response to stroma**. Fold change lists were generated using Genesping (both used fold change lists of 1.1 fold and p < 0.05) for both the primary (blue) and cell line (red) arrays. The gene lists were compared using Entrez gene IDs to find common upregulated (A) and downregulated genes (B).

**Figure 6:**
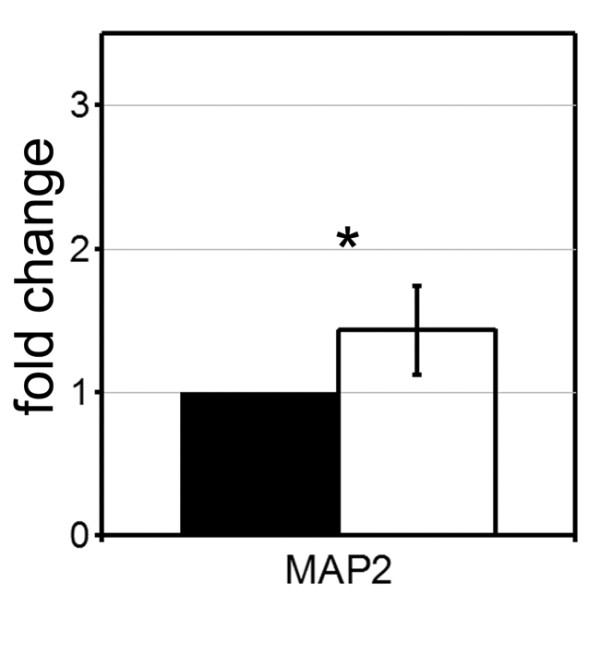
**Confirmation of MAP2 expression**. QRT-PCR was performed on mRNA isolated from 3D BPH-1 acini cultured for 7 days with (white) or without stroma (black) from three different patients (in duplicate). The average fold difference in gene expression was expressed relative to growth without stroma ± standard error ** P < 0.05.

To aid the identification of genes that are most relevant to human adult tissue we directly compared the gene lists from the microarray analysis of primary cells to those from the microarray analysis of cell line, this identified 36 genes which were upregulated in both lists and 45 genes that were down regulated (Figure 5). Interestingly, only three genes from tables 2 and 4 describing the highly differentially expressed genes in either model appeared in this figure (KRT6B, TOP1 and GNG7) and none of these genes have a known function relating to morphology. To identify genes most likely to have a function in morphology or adhesion, the gene ontology (GO) molecular function and cellular component terms were found for each gene and then we identified all genes which contained the phrases 'TGF beta', 'E-cadherin', 'tight junctions', 'actin', 'cytoskeleton', 'cell shape', 'cell adhesion'. Several gene groups were identified: actin binding (GO:0003779), FHOD3, ABLIM1, TMOD4, MYH10; actin cytoskeleton organisation (GO: 0030036), DIAPH2, FHOD3; regulation of Rho signal transduction (GO:0035023), BCR; regulation of cell shape (GO:0008360), MYH10; cell morphogenesis (GO:0000902), STK4; microtubule (GO:0005874), MAP2, KIFC1; cell-matrix adhesion/cell adhesion (GO:0007160, GO:0007155), NID2, CD44, ITGA6. In addition we identified a large group of genes associated with ion channel/ion transporter activity (GO:0005244, GO:0005216, GO:0015081, GO:0046873), CACNA1C, CACNB2, KCNH2, SLC8A1, SLC39A9. The remaining genes were predominantly associated with transcription, metabolism or protein transport. We further identified genes associated with developmental signalling pathways, using GO terms or literature searches, this identified; POFUT1, (notch signalling); IRX2, HOXA5 (homeobox genes); FZD2 (Wnt signalling); FGF11, SOX4 (TGF beta) and SMARCC1. All these developmental pathways have known and complex roles in prostate development or in the remodelling of epithelial sheets, their function within our model remains to be determined [18,19]. Importantly SOX4 is associated with TGF beta signalling [20] though it was not listed within the Kegg pathway. Stromal function was confirmed by the down regulation of CD44, ITGA6 and KRT6, downregulation of these genes is associated with epithelial differentiation, a known role of stroma [21,22]. MAP2 was chosen to validate the list of common genes. Using QRT-PCR we confirmed that BPH-1 cells cultured in the presence of stroma (different stromal mix to either array) had upregulated MAP2 expression (Figure 6).

## Discussion

This research highlights the difficulties faced by a cell biologist trying to select the most appropriate model system for their research. In our work we prefer to validate all our experiments using primary cultures to ensure our research reflects human biology and disease. The use of a single cell line for experiments is common because they provide a reliable and repeatable model. However cell lines often suffer from 'genetic drift' in long term culture and do not reflect the tissue from which they were derived nor their original architecture and can often provide inadequate data [23]. Experimentation on a panel of cell lines should be adopted to demonstrate that a result holds true across many models and not just one particular laboratory model. However, as demonstrated here, the use of a wider range of cell models reduces our ability to find valid genes from a microarray analysis. Indeed, multiple testing correction was not applied to the primary microarray because this produced no significant genes, due to the large heterogeneity between patient samples of primary epithelia and stromal cells. Such heterogeneity is common place when working with human tissues [24,25]. The problems produced by heterogeneity can be reduced by increasing the number of samples. Primary cultures are technically difficult to produce and take time to acquire. Rather than increase our sample size, which was already considerable, we decided to compare our data to that of a cell line model and combine several stromal cultures into one (to reduce the heterogeneity from the stroma). By combining the two microarray analyses and analysing common pathways as well as individual genes, we hope to identify tissue relevant genes in a cell line. These genes may also have more fundamentally importance to the mechanism of increased adhesion. Future work will seek to provide functional confirmation of the identified genes and pathways and confirm that the genes have the same function within the cell line and the primary models and whether this relates to normal tissue. At this point the work remains preliminary until future functional studies are carried out.

Using 3D cultures to model the stromal maintenance of adult epithelial tissues, we previously discovered that stromal cells signal to increase the lateral cell adhesions of epithelial cells [7]. This is an intriguing discovery since in monolayer epithelial cells are well known to scatter in response to stroma or stromal conditioned media [26]. Examination of the genes that were highly upregulated or highly down regulated during this morphological change by two arrays of primary and cell line models showed little agreement. Only KRT6 was highly down regulated in both. Examination of the significant pathways identified only TGF beta signalling, and further examination of the genes within the pathway identified only one, SOX4, to have common expression. One interpretation of these results is that there is poor agreement between the models and this is certainly true but the genes identified in common are likely to be more relevant and more fundamental to the processes under study. Analysis of common genes identified important morphological clustering of genes, with the following terms; actin binding, cytoskeleton, ion channel/ion transporter activity and genes associated with developmental pathways. The present knowledge of these genes with particular reference to morphology will be discussed.

The importance of TGF beta signalling has confirmed our earlier observation that stromal derived TGF beta is important for the control of lateral epithelial cell adhesions (paper in submission). SOX4 is an important transcription factor in development and interacts with many morphology related pathways, (Hedgehog, Notch and Wnt). SOX4 stabilises β-catenin protein and enhances β-catenin/TCF activity [20]. Over-expression of SOX4 is associated with many cancers (including prostate) and anchorage independent growth [27]. The association of increased adhesion with down-regulation of SOX4, found here, is an important mechanism to study further. Other genes associated with TGF beta signalling were identified from the list of common genes, these were STK4, ITGA6 and CILP2. STK4 is an important signal transducer for the TGF beta family [28]. TGF beta induced down regulation of ITGA6 and upregulation of CILP2 (cartilage intermediate layer protein 2) has already been demonstrated in other tissue models [29,30]. Therefore these genes may provide good candidates with which to test the importance of TGF beta signalling in our model and also the importance of stromal derived TGF beta.

Actin binding and cytoskeleton genes provided the most likely set of genes to have a role in adhesion. We found up regulation of MAP2 (microtubule-associated protein 2), which is a major regulator of microtubule dynamics and is best known for its role in neuronal development [31]. MAP2 is also located in non-neuronal cells where its biological functions remain largely unknown, it can interact with F-actin and increase cellular migration by inducing microtubule bundling at the cell periphery [32]. Microtubules are known to contact adherens junctions and are required (alongside myosin II) for cadherin junction formation [33]. Microtubules may prove to be an important area for future focus due the further identification of KIFC1, a kinesin/microtubule motor protein with important functions in polarity and cell division [34]. Several actin associated genes were found including DIAPH2 (Diaphanous 2), FHOD3 (formin homology 2 domain 3), BCR (breakpoint cluster region), ABLIM1 (actin binding LIM protein 1), MYH10 (non-muscle myosin heavy chain IIB or NMII) and TMOD4 (tropomodulin 4). Diaphanous proteins localise to cell to cell contacts where they also play an important role in cadherin junction formation [35-37]. Formin homology 2 sequences are essential to induce actin assembly, but also inhibit actin elongation [38]. BCR can regulate the activity of Rho-like GTPases and is thought to regulate signalling pathways at the sites of cellular junctions [39,40]. ABLIM1 has uncertain biological function, but it may act as a scaffold protein [41]. MYH10 has a fundamental role in processes that require cellular reshaping and movement. NMII uses actin cross-linking and contractile functions to regulate the actin cytoskeleton. It has complex roles in migration, polarity and the formation and promotion of stable cell-cell junctions [42]. Crucially, NM II-driven mechanisms also govern the three dimensional organization of epithelial tissues, studied in *X. laevis *and *D. melanogaster *during early embryonic development and organogenesis. Therefore upregulation of MY10 found here may promote polarity and adhesion. TMOD4 is an actin filament capping protein that maintains the length of the actin filaments in skeletal muscle and in has a role in cell membrane dynamics [43,44]. None of these cytoskeletal genes have known functions associated with the prostate.

Several calcium and potassium channels were up regulated on both arrays (CACNA1C, CACNB2, KCNH2, SLC8A1) these may provide a means of modulating cell junctions by controlling the intracellular levels of calcium and potassium [45]. Recent bioinformatic and proteomic analysis of epithelial tight junctions revealed that synaptic proteins and signalling molecules were associated with tight junctions, and these included potassium and calcium voltage gated channels [46]. The authors suggested that tight junctions may have a novel role as an epithelial synapse for cell to cell communication. Validation of these results may provide further insights into this hypothesis.

Before this study, tight junctions and adherens junctions were likely candidates to be involved in increased cell to cell adhesion. They are dynamic structures linked to the acto-myosin cytoskeleton and are regulated by Rho/Ras-GTPases [19]. Microarray analysis did not indicate a clear role for either junction. Junctional pathways and genes were associated with either primary or cell line model but not both. Since adhesion depends on the interaction of junctional proteins with the cytoskeleton, our combined analysis indicates a greater role for the cytoskeleton and its regulators over that of junctional proteins in adhesion. This is an important finding which may have been missed without combining the models and will be important to prove further. Significantly MYH10 can regulate the assembly of apical junction complexes and increase the height of lateral cell domains [47], its inhibition reduces tight junctions and adherens junction formation [47-50]. Both tight junctions and adherens junctions are controlled by the TGF beta superfamily. The effect of TGF beta on adhesion varies according to the experimental model, making a strict interpretation difficult. TGF beta can antagonise tight junction formation in cell lines [51,52] but increase barrier function through upregulation of claudins in tissue [53]. Although TGF beta is known to initiate epithelial-mesenchymal transition it can also promote adhesion by targeting E-cadherin to the cell membrane via the ELF adaptor protein (ELF4 was highly upregulated by both arrays) [54].

## Conclusions

We used microarray analysis and bioinformatics to identify candidate epithelial genes which control lateral cell adhesion under stromal stimulation. We confirmed the importance of TGF beta signalling, and in particular SOX4. Analysis of genes that were common to both cell line and primary arrays found several morphology related gene clusters; actin binding, GTPase activator activity, cytoskeleton, protein binding, proteinaceous extracellular matrix, ion channel/ion transporter activity and genes associated with developmental pathways. These candidates will be investigated in future functional studies. This work highlights the complexity of any biological process and the value of combining gene array data from different models to identify important pathways and genes. Overall we have shown the complexity of stromal controlled epithelial morphology. The study of intercellular adhesion is a fast expanding field, and our identification of genes associated with actin binding, microtubules and anion signalling complements newly emerging ideas.

## Competing interests

The authors declare that they have no competing interests.

## Authors' contributions

KC participated in the design of the study, performed data analysis and design, prepared the primary culture samples and coordinated the Operon array study. JFP prepared samples for Affymetrix array and performed qRT-PCR assays. MG and CK coordinated and performed the Operon array. NA coordinated the Affymetrix array and was involved in data analysis. DP was involved in data analysis. SHL conceived of the study, participated in study design and co-ordination, analysed experiments and wrote the manuscript. All authors read and approved the final manuscript.

## Supplementary Material

Additional file 1 **Table S1: Primers and probes for QRT-PCR **primer sequences. Click here for file
