# Supplementary material for: Stromal upregulation of lateral epithelial adhesions: Gene expression analysis of signalling pathways in prostate epithelium
Source: J Biomed Sci. 2011 Jun 22;18(1):45. doi: 10.1186/1423-0127-18-45 (PMC3141633; doi:10.1186/1423-0127-18-45)
Supplement: Additional file 1 — Table S1: Primers and probes for QRT-PCR primer sequences. [file 1423-0127-18-45-S1.DOC]

**Table S1: Primers and probes for QRT-PCR**

|  | Forward | Reverse | Probe |
| --- | --- | --- | --- |
| MAP2 | 5'-CCCGACGACTCAGCAATG-3' | 5'-AAGTGGCAAGCTGAGGAGATTC-3' | 5'-TCCTCGTCTGGAAGCATCAACCTGC-3' |
| HPRT | 5'-GCTCGAGATGTGATGAAGGAGAT-3' | 5'-AGCAGGTCAGCAAAGAATTTATAGC-3' | 5'-CCATCACATTGTAGCCCTCTGTGTGCTC-3' |
